# Construction of Multi-Scale Consistent Brain Networks: Methods and Applications

**DOI:** 10.1371/journal.pone.0118175

**Published:** 2015-04-13

**Authors:** Bao Ge, Yin Tian, Xintao Hu, Hanbo Chen, Dajiang Zhu, Tuo Zhang, Junwei Han, Lei Guo, Tianming Liu

**Affiliations:** 1 Key Laboratory of Modern Teaching Technology, Ministry of Education, Xi’an, China; 2 School of Physics & Information Technology, Shaanxi Normal University, Xi’an, China; 3 Department of Communication, Xi’an Communications Institute, Xi’an, China; 4 School of Automation, Northwestern Polytechnical University, Xi’an, China; 5 Cortical Architecture Imaging and Discovery lab, Department of Computer Science and Bioimaging Research Center, The University of Georgia, Athens, GA, United States of America; Beijing Normal University, CHINA

## Abstract

Mapping human brain networks provides a basis for studying brain function and dysfunction, and thus has gained significant interest in recent years. However, modeling human brain networks still faces several challenges including constructing networks at multiple spatial scales and finding common corresponding networks across individuals. As a consequence, many previous methods were designed for a single resolution or scale of brain network, though the brain networks are multi-scale in nature. To address this problem, this paper presents a novel approach to constructing multi-scale common structural brain networks from DTI data via an improved multi-scale spectral clustering applied on our recently developed and validated DICCCOLs (Dense Individualized and Common Connectivity-based Cortical Landmarks). Since the DICCCOL landmarks possess intrinsic structural correspondences across individuals and populations, we employed the multi-scale spectral clustering algorithm to group the DICCCOL landmarks and their connections into sub-networks, meanwhile preserving the intrinsically-established correspondences across multiple scales. Experimental results demonstrated that the proposed method can generate multi-scale consistent and common structural brain networks across subjects, and its reproducibility has been verified by multiple independent datasets. As an application, these multi-scale networks were used to guide the clustering of multi-scale fiber bundles and to compare the fiber integrity in schizophrenia and healthy controls. In general, our methods offer a novel and effective framework for brain network modeling and tract-based analysis of DTI data.

## Introduction

Construction and modeling of structural and functional brain networks has gained great interest recently, due to its significant importance in revealing the brain’s structural architecture and functional dynamic behaviors [1–4]. In general, the whole-brain anatomical and/or functional connectivity in living humans can be modeled as networks via diffusion magnetic resonance imaging (dMRI) and/or functional MRI (fMRI) data. Typically, network nodes are associated with distinct gray-matter regions of interest (ROIs), and edges are usually defined as morphological correlation [5], functional correlations [6] or fiber pathways [2] between cortical ROIs. Then, a variety of graph theory metrics could be applied to reveal the brain’s network properties in different applications [7,8].

Generally speaking, previous methods in defining network nodes to construct whole brain structural or functional networks can be roughly classified into three broad categories. The first group of methods divided the brain into nodes according to an existing anatomical brain atlas [5,9,10]. These methods typically depend on the brain atlas used and image registration techniques. The second category determined nodes in a more user-defined manner, for example, the authors in [2] and [11] selected voxels as seeds, and merged the neighbor voxels repeatedly until a predefined size of region appeared. Some other researchers directly took each voxel as node to compute the functional or structural connectivity between them [12–14]. The third school of methods is data-driven, which uses morphological [15], structural [16] or functional features [17] to define nodes. These methods do not depend on specific models, and have the optimal division within individual brains.

Recently, some research studies indicated that brain network is a hierarchical modular organization [18,19]. Several simulation studies have also demonstrated that the structural hierarchical modular organization determines the functional hierarchical characteristics in the brain [20,21], and functional clusters coincide with the anatomical communities at different scales [6]. These previous studies suggested that brain networks are multi-scale and modular, so our aim of constructing such a multi-scale structural brain network is valuable and consistent with the existing neuroscience findings, which has not been sufficiently addressed by the above brain network constructing methods. Other significance of our work lies in that we can perform interesting applications such as analyzing and measuring brain network characteristics in the appropriate scales [22] because neuroimaging-based measurements might be sensitive to different scale, given that different brain diseases or cognition occur at different spatial scales [23]. Essentially, the constructed brain networks at different scales should be truly multi-scale, that is, having a nested relationship. Furthermore, it has been pointed out [4] that the determination of nodal size of brain network, inference of the optimal number of spatial scales and the appropriate number of clusters in each scale are all critically important and that it is quite difficult to construct appropriate brain networks. The second problem we would address is establishment of the correspondences of multi-scale brain networks across individuals and populations, which is remarkably difficult because of the limitation of current brain image registration algorithms in dealing with the remarkable variability of brain structures. Without the correspondences between those multi-scale brain networks, we cannot effectively compare brain networks across individuals [23] and many meaningful statistical measurements of network behaviors cannot be accurately performed. To address these two challenges that have not been sufficiently addressed by the above previous methods in defining network nodes to construct brain networks, this paper aims to present a novel approach to constructing multi-scale common structural brain networks with intrinsically-established correspondences across individuals and populations.

Recently, we developed and publicly released the DICCCOL (Dense Individualized and Common Connectivity-based Cortical Landmarks) system [24]. These 358 group-wise consistent DICCCOL landmarks have been shown to possess intrinsically-established structural and functional correspondences across individuals [24]. Importantly, these 358 landmarks can be accurately predicted in each individual brain with DTI data [24] and have been well reproduced in over 240 individual brains in our previous experiments [24]. This indicates that these 358 landmarks offer a universal and individualized brain reference system. Thus, these DICCCOL landmarks can be used as the corresponding nodes for brain network construction. In this paper, by treating the DICCCOL landmarks as the primitive network nodes, we adopt a local geodesic distance and the shortest path length as a combined feature to divide the primitive nodes into sub-networks (nodes) at multiple scales using an improved effective multi-scale spectral clustering algorithm [25]. The premise behind it is that human brains are likely to maximize the cost efficiency of parallel information processing in large-scale networks [26,27]. That is, the brain tends to increase its efficiency (favoring a high density of short-range local connections) while decreasing the cost (favoring the selection of a few long-range fiber connections mediating efficient information transfer between spatially distributed regions), like an economical small-world network [5]. Therefore, we designed two features, that is, the geodesic distance on cortical surface and the shortest path length on fiber connection graph between landmarks, which contain both short-range and long-range connections information, and combined them as an input for the multi-scale spectral clustering algorithm. As a result, the optimal scales and sizes/numbers of nodes are determined by the effective features and the multi-scale spectral clustering. Given that DICCCOL landmarks possess intrinsic structural correspondences across individuals, these brain networks possess intrinsically-established correspondences across multiple scales and across individual brains. We performed a simple group-wise inference of corresponding brain sub-networks by averaging the input feature matrices among subjects, thus addressing the above-mentioned second challenge. Extensive experimental results have demonstrated that the methods can robustly generate reproducible multi-scale consistent and common structural brain networks. As a practical application, the multi-scale networks were used to guide the clustering of multi-scale fiber bundles and to examine the fiber integrity in schizophrenia and healthy controls. Notably, an early short version of this computational framework was presented in the IPMI conference [28].

## Materials and Methods

**Ethics Statement:** For dataset 1, written informed consent was obtained from each participant, and all procedures were approved by the IRB of Beijing Normal University Imaging Center for Brain Research. For dataset 2, nine healthy young adults were recruited at The University of Georgia (UGA) under UGA IRB approval. All participants provided their written informed consent to participate in this study and the study was approved by UGA IRB. Dataset 3 was downloaded from the NA-MIC data source which was acquired under IRB approvals (http://hdl.handle.net/1926/1687). The study was approved by the Brigham and Women’s Hospital IRB. Participants provided their written informed consent to allow that their clinical records can be used in research studies including this one.

### Overview

**Fig 1:**
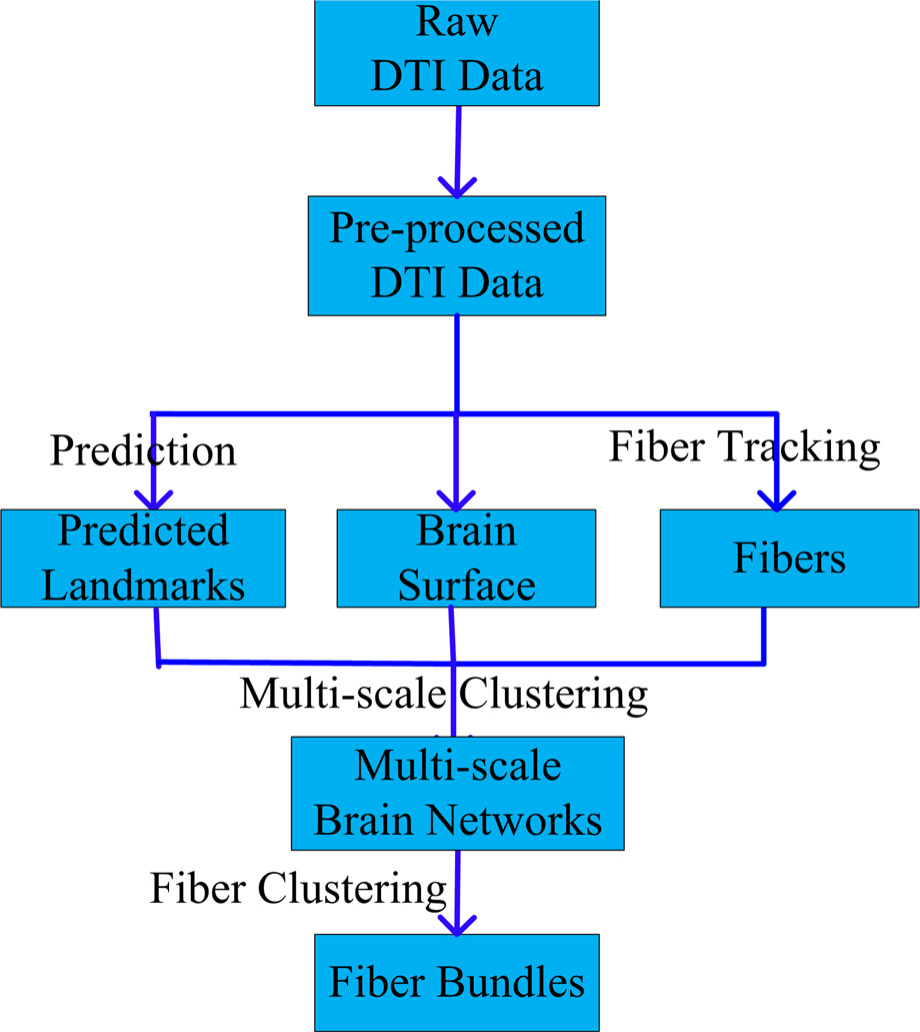
Flowchart of the proposed computational framework.

As summarized in Fig 1, our multi-scale common brain network construction framework includes the following steps. First, we pre-processed the raw DTI data and performed whole-brain streamline fiber tracking and surface reconstruction based on the processed DTI data. Then, we predicted the 358 consistent DICCCOL landmarks for all subjects via the methods in [24]. After that, we computed the geodesic distance and the shortest path length between landmarks based on the cortical surface and fiber information. Then an improved multi-scale spectral clustering algorithm [25] was applied to derive the optimal number of clusters within each scale and the optimal number of scales automatically, resulting in the multi-scale brain networks. Finally, based on these networks, we clustered the whole-brain streamline fibers into multi-scale corresponding bundles across individuals.

### Data Acquisition and Pre-processing

Dataset 1: 100 healthy adults (the average age was 21.2) from the publicly released dataset by the Beijing Normal University, China [29]. The DTI data were acquired using a SIEMENS TRIO 3-T scanner with single-shot Echo-Planer Imaging-based sequence. The parameters were as follows:49 axial slices, 2.5 mm slice thickness, a TR of 7200ms, a TE of 104ms, 64 diffusion directions with b-value 1000s/mm^2^, a matrix size of 128×128, and a FOV of 230×230mm^2^. Additional details of the dataset are referred to Yan et al., 2011.

Dataset 2: Nine healthy young adults (the average age was 25.8) recruited at The University of Georgia (UGA) under UGA IRB approval were scanned in a GE 3T Signa MRI system (GE Healthcare, Milwaukee, WI) using an 8-channel head coil at the UGA Bioimaging Research Center (BIRC), Athens, GA. Diffusion tensor imaging (DTI) data were acquired with the following parameters: an isotropic spatial resolution of 2mm×2mm×2mm, a FOV of 250×256 mm^2^, a TR of 15.5s and TE min-full, a b-value of 1000 with 30 DWI gradient directions and 3 repetitions of b = 0.

Dataset 3: 10 SZ patients and 10 healthy control subjects (the average age was 42.7) were downloaded from the NA-MIC dataset (http://hdl.handle.net/1926/1687) and used for application. Briefly, DTI scans were acquired on a 3 Tesla GE system using an 8 Channel coil and Array Spatial Sensitivity Encoding Techniques (51 directions with b = 900s/mm2, 8 baseline scans with b = 0, TR = 17s, TE = 78 ms, FOV = 24×24 cm^2^, matrix size = 144×144, 1.7 mm slice thickness with 85 slices to cover the whole brain, voxel size 1.67×1.67×1.7mm^3^). Two cases of SZ subjects were excluded due to the low quality of DTI tractography and cortical surface reconstruction.

Pre-processing of the DTI data included brain skull removal, motion correction, and eddy current correction by FSL. After the pre-processing, brain tissue segmentation was performed on the DTI data via the approaches in [30]. Based on the brain tissue map, the gray matter (GM)/white matter (WM) cortical surface reconstruction was performed [31]. The streamline fiber tracking was performed using the MEDINRIA (http://www-sop.inria.fr/asclepios/software/MedINRIA) (FA threshold: 0.2; minimum fiber length: 20). Then, the DICCCOL landmarks were predicted according to the steps in [24]. Finally, in order to find all fibers connecting cortical landmarks, we prolonged or shortened the fibers to make them reach the gray matter cortex according to the method in [32,33], Then we can easily identify the fibers connecting cortical landmarks by judging if the two end segments of each fiber have point of intersection with the cortical landmark.

### Constructing Multi-scale Common Brain Networks via DICCCOLs

We aimed to construct multi-scale common brain networks based on DICCCOL landmarks by means of a top-down clustering procedure. First, an affinity matrix was obtained by measuring the similarity between landmarks as defined below. Then, a multi-scale brain networks was calculated by iterative partition based on the graph defined by the affinity matrix. That is, by treating all landmarks as one node initially, we clustered these landmarks into sub-networks according to the similarities between landmarks, and thus these sub-networks can be viewed as nodes at the next scale. For each node, we repeated the partition step iteratively, finally forming a group of multi-scale brain networks.

### The Similarity Measure of Landmarks

As stated in the introduction section, we employ short-range and long-range connection information to design two features, that is, the geodesic distance on cortical surface and the shortest path length on fiber connection graph between landmarks, and their combination will be used as the input for the multi-scale spectral clustering algorithm. Since those long-range fiber connections can be tracked, while the local short-range connection cannot be obtained by current DTI and fiber tracking techniques, we have to seek alternative methods to represent the possible local connections. Here, we used the geodesic distance [34] to measure the local affinity and divide the brain into the separate regions, in that those cortical regions with small geodesic distance are apt to connect each other by short-range connections. Meanwhile, we integrated spatially separated regions by long-range fiber connections. By means of combining these two features, it is expected that a reasonable brain network can be constructed.

**Fig 2:**
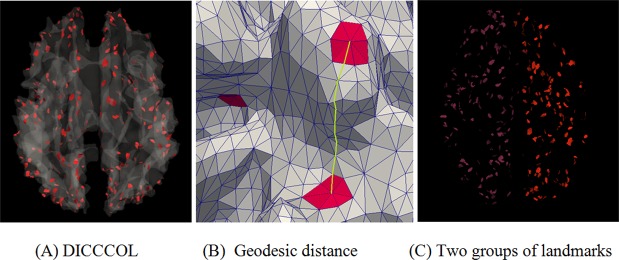
Illustration of DICCCOLs and geodesic distance. (A) 358 DICCCOLs represented by red patches. (B) An illustration of the shortest geodesic path between two landmarks (yellow line). (C) Bi-partitioning of DICCCOLs (purple and red) based on geodesic distance using spectral clustering algorithm.

Based on the reconstructed cortical surface, the geodesic distance, represented as *G*_*n*_(*S*_*i*_, *S*_*j*_) between any pair of landmarks, measures the length of the shortest geodesic path from *S*_*i*_ to *S*_*j*_ on the cortex of subject *n*, where *S*_*i*_ is the location of the *i* ^*th*^ landmark. For the purpose of computational efficiency, the approximation solution algorithm [34] for geodesic path on triangle meshes was adopted to compute the shortest distance between DICCCOL landmarks on cortical surfaces. Fig 2(B) shows an example of a shortest geodesic path from one landmark to another. Because DICCCOL landmarks locate on the remarkably folded cortical surface and it is desirable to cluster landmarks that are likely to have local connections, geodesic distance, instead of using Euclidian distance, is more preferable. To further demonstrate, we applied the baseline spectral clustering algorithm [35] to bi-partition DICCCOLs based on geodesic distance. As shown in Fig 2(C), the landmarks were divided into the left and right parts, which are correspondent to the two hemispheres. This result is in agreement with current neuroanatomical knowledge.

However, the nodes resulted from the geodesic distance are probably not an optimal division in term of fiber connection and functional integration. That is, two landmarks that belong to two different sub-networks divided by geodesic distance are probably more appropriate to be treated as one node if they have a strong fiber connection. Moreover, literature studies have demonstrated that those brain regions connecting each other with fibers tend to have a higher functional coherence [36]. Thus, if we consider the fiber connection information simultaneously, the result would be expected to be different from that of the geodesic distance used only.

**Fig 3:**
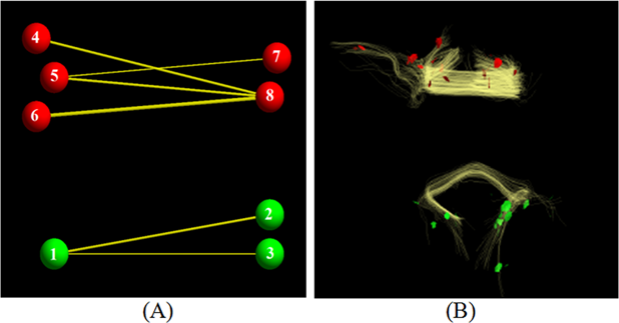
Examples of two sub-networks. (A) An illustration that how these landmarks forming a sub-network. (B) A real example that two group of landmarks which have strong connectivity within each group form two sub-networks.

In this work, we intend to group those landmarks with strong fiber connections into one sub-network. In order to achieve this, we first computed the fiber connection matrix *C*_*n*_(*s*_*i*_,*s*_*j*_) of landmarks, which indicates whether there is a direct fiber connection between landmarks. Then we computed the shortest path length *P*_*n*_(*S*_*i*_,*S*_*j*_)between *S*_*i*_ and *S*_*j*_ as another feature to represent the affinity between nodes. *P*_*n*_ and *C*_*n*_ are computed as follows:

$$P_{n}\left(S_{i},S_{j}\right)=Dijkstra\left(C_{n}\left(S_{i},S_{j}\right)\right)$$ (1)

$$C_{n}\;\left(S_{i},\;S_{j}\right)=\{\begin{array}{cc} 1 & if\;the\;number\;of\;fibers\;between\;landmarks\;>\;thres\\ 0 & \;\;\;\;\;\;\;\;\;\;\;\;\;\;\;\;\;\;\;\;\;\;\;\;\;\;\;\;\;\;\;\;\;\;\;\;\;\;\;\;\;\;\;\;\;\;\;\;\;\;\;\;\;\;\;\;\;\;\;\;\;\;\;\;\;\;\;\;\;\;\;\;\;\;\;\;\;\;\;\;\;\;\;\;\;\;\;\;\;\;\;\;\;\;\;\;else \end{array}$$ (2)

where *C*_*n*_(*s*_*i*_,*s*_*j*_) is a binary judgment, in which 1 denotes that there is strong direct fiber connection between two landmarks. *p*_*n*_(*S*_*i*_,*s*_*j*_) denotes the shortest path length from the landmark *S*_*i*_ to *S*_*j*_of the *n*-th subject, and was computed from *C*_*n*_(*S*_*i*_,*S*_*j*_) by the Dijkstra shortest path algorithm [37]. It is the number of hops from *S*_*i*_ to *S*_*j*_, where each direct fiber connection represents a hop, for example, *P*_*n*_(*S*_*i*_,*S*_*j*_) = 1 if the two nodes have direct fiber connection, and *P*_*n*_(*S*_*i*_,*S*_*j*_) = *m* if they connect each other indirectly and via *m* hops. Apparently, the two nodes with less hops of fiber connections are more likely to belong to the same sub-network, so *P*_*n*_(*S*_*i*_,*S*_*j*_) measures how likely two landmarks belong to the same node. The smaller *P*_*n*_(*S*_*i*_,*S*_*j*_) is, the more likely the two belong to the same node. As an example, Fig 3(A) illustrates that those landmarks that have direct or indirect fiber connection can be clustered into the same sub-network, if they have the small shortest path length, although some landmarks have no direct fiber connections. It will be preferable to cluster these landmarks into the same cluster in real situation. For instance, the shortest path length between landmark 2 and 3 is 2, and the shortest path length between landmark 6 and 7 is 3, then the shortest path lengths between them are relatively small. Fig 3(B) provides a real example that landmarks are clustered into two groups. Here, only a small number of landmarks are selected to be clustered, and it is assumed that the original spectral clustering algorithm [35] is adopted to bi-partition these landmarks. As shown in Fig 3(B), two groups of landmarks were obtained, forming two sub-networks (red and green), within each of them. The shortest path lengths between these landmarks are relatively small and the connectivities are relatively high. This simple example illustrates that the measurement can reasonably group the DICCCOL landmarks.

By considering each DICCCOL landmark as the node of an undirected weighted graph and each pair of connected landmarks by an edge, we combined the two distances described above to compute the edge weight W_ij_(δ) according to the following formula:

$$\left\{ \begin{array}{l} {\text{W}}_{ij}\left(\delta \right)=\exp\left(-\frac{{\left(\alpha \times \text{G}\left(S_{i},S_{j}\right)+\left(1-\alpha \right)\times \text{P}\left(S_{i},S_{j}\right)\right)}^{2}}{\delta^{2}}\right)\\ \text{G}\left(S_{i},S_{j}\right)=\sum_{n=1}^{N}\frac{{\text{G}}_{n}\left(S_{i},S_{j}\right)}{\max\left({\text{G}}_{n}\left(S_{i},S_{j}\right)\right)}/N\\ \text{P}\left(S_{i},S_{j}\right)=\sum_{n=1}^{N}\text{histEq}\left(\frac{{\text{P}}_{n}\left(S_{i},S_{j}\right)}{\max\left({\text{P}}_{n}\left(S_{i},S_{j}\right)\right)}\right)/N \end{array}\right.$$ (3)

**Fig 4:**
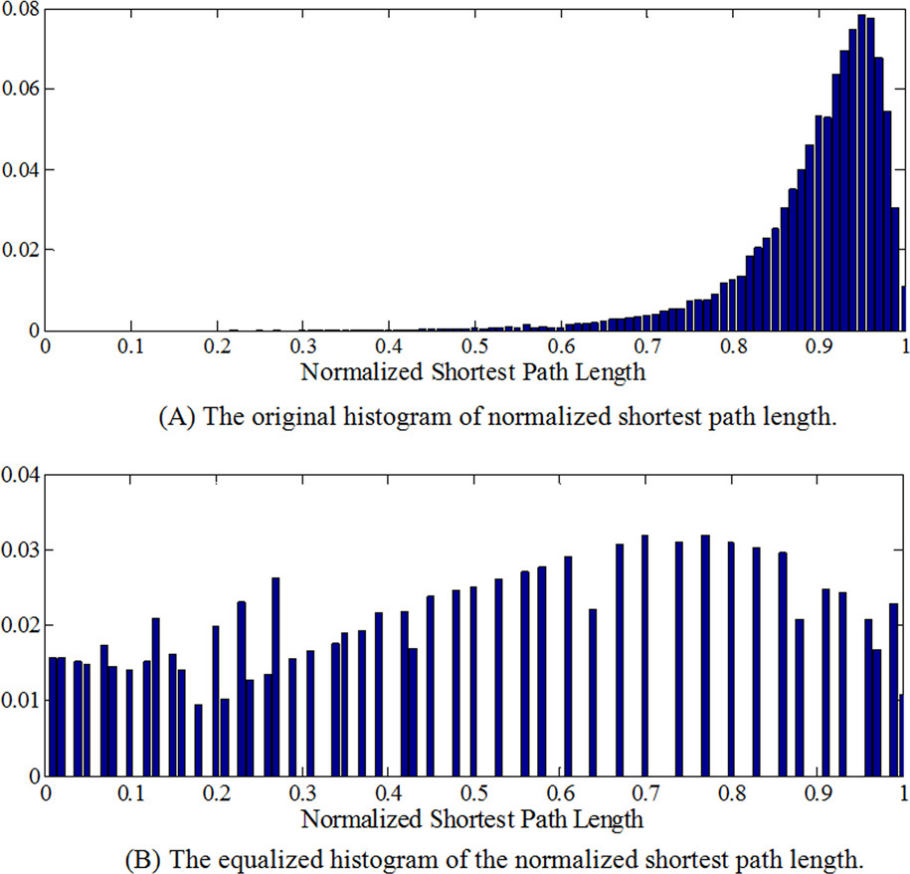
The histograms before and after equalization.

Where δ is the length scale of the Gaussian similarity function, N is the number of subjects. The smaller it is, the smaller is the ‘neighborhood’ of a landmark [25]. In order to combine *G* and *P* efficiently, they need to be normalized to be in the same range [0, 1], and *P*_*n*_(*S*_*i*_,*S*_*j*_) is histogram-equalized to make the original concentrated distribution of the *P*_*n*_ more uniform and distinguished in the range [0, 1]. Thus we made *P* contribute to *W* more efficiently. Furthermore, in order to keep the correspondences across individuals and obtain the group-wise results, we averaged the geodesic distance and the shortest path length among subjects. This averaged connectivity matrix ensured us to eliminate inter-individual differences and obtain group-wise results. Fig 4 shows the histograms before and after equalization, in which we can see that after equalization the concentrated distribution part of the shortest path length was stretched, which makes the value more distinguished. α is a tradeoff coefficient between the short-range(*G*) and long-range connections information(*P*). Here, we have tested the different tradeoff coefficients (α) and compared the corresponding results. It was found that the resulting sub-networks are similar and more consistent with the existing knowledge when α = 0.4~0.65, thus we selected α = 0.5.

**Fig 5:**
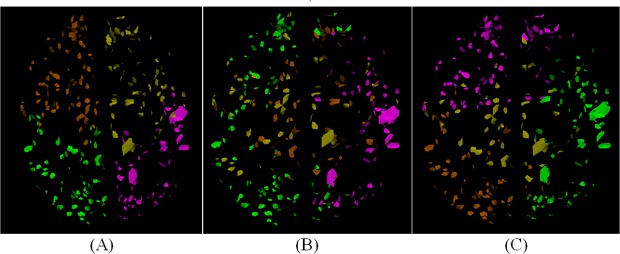
The 4 nodes clustered by using *G* ^*2*^, *P* ^*2*^ and ((*G+P*)/2)^2^ respectively if we suppose that all landmarks will be clustered into 4 groups. The colors of nodes were randomly assigned. If we check (A) resulted from using geodesic distance (*G* ^*2*^) only, those landmarks close in spatial space are divided into one group. While in (B), all landmarks were divided by using fiber connection information (*P* ^*2*^) only, and those landmarks with strong fiber connection were clustered into one group. The result of these two feature’s concurrent effect is exhibited in (C), which looks like a trade-off between (A) and (B).

We compared the clustering results of adopting geodesic distance (*G*), shortest path length (*P*) and their combination, accordingly, by using *P* ^*2*^, *G* ^*2*^ and (0.5×*G*+0.5×*P*)^2^ to compute *W*. It is supposed that all landmarks were clustered into the four nodes and the original spectral clustering algorithm [35] was adopted here. Fig 5(A) shows the 4 nodes which were divided by geodesic distance alone, while Fig 5(B) gives the 4 nodes divided by the shortest path length alone. We can find that the landmarks within each group in (A) are close neighbors in spatial location, while the landmarks belonging to the same group in (B) are not necessarily the case, because one feature separates the cortex according to the spatial distance, while another integrates brain landmarks into sub-networks according to fiber connection. Therefore, these two features both play a part in our understanding of formation of node, the result of their concurrent effect is exhibited in Fig 5(C), which looks like a tradeoff between (A) and (B) and the combined feature is the preferred way to construct brain network in this paper.

### Multi-scale Clustering

**Fig 6:**
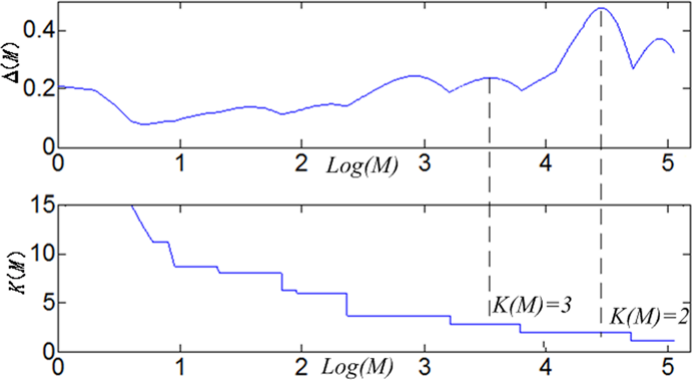
The illustration for basic random walk spectral clustering.

The edge values W_ij_(δ) associated with the graph were then feed to a clustering method to group these landmarks into sub-networks. In general, traditional spectral clustering methods need to be specified with different parameters. As a consequence, the clustering results may be subject to variation and uncertainty. In contrast, the multi-scale spectral clustering algorithm [25] can automatically explore the data structures (that is, learn the optimal δ) and infers different plausible values for the number of clusters (K). It tries to seek a reasonable partition of the graph such that the random walk stays within the same cluster and seldom jumps between different clusters from a random walk perspective. The algorithm results in a tree structure partition of data points, starting by partitioning in a large scale, and then recursively partitioning each of the obtained sub-trees. Fig 6 illustrates how the basic random walk spectral clustering determines the optimal number of clusters using the following functions:

$$\Delta \left(\text{M,}\delta \right)=\max_{\text{k}}\left(\lambda_{\text{K}}^{\text{M}}\left(\delta \right)-\lambda_{\text{K+1}}^{\text{M}}\left(\delta \right)\right)$$ (4)

$$\text{K}\left(\text{M,}\delta \right)=\arg\max_{\text{k}}\left(\lambda_{\text{K}}^{\text{M}}\left(\delta \right)-\lambda_{\text{K+1}}^{\text{M}}\left(\delta \right)\right)$$ (5)

Here, λ_K_ is the kth eigenvalue, and $\lambda_{\text{K}}^{\text{M}}$ is λ_K_ to the Mth power. The bigger Δ(M, δ) is, the more distinguished is the structure revealed by it after a random walk of M steps. Here, δ is fixed. So, Δ(M, δ) ≈0.45 is maximal in Fig 6, and its corresponding K = 2. That is, it should be clustered into 2 groups here.

**Fig 7:**
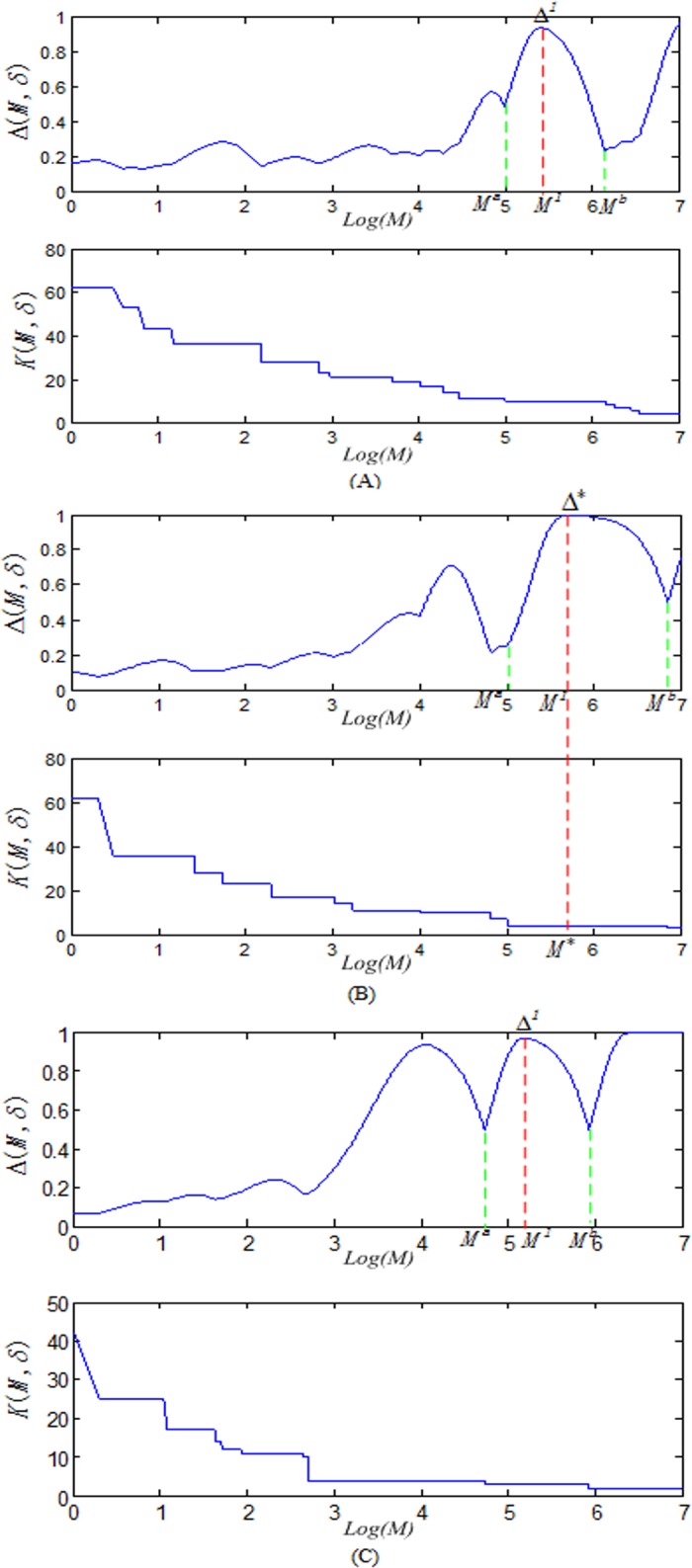
Automatically derive the parameters δ and K according to the curve of the Δ(M,δ) and Δ(M,δ). First, for each δ in (A), (B) and (C), chose the M^1^ with the highest Δ (denoted as Δ^1^), then selected the(δ*,M*) and corresponding K with the highest stability measure α = M_b_-M_a_ among all δ. We can see that δ* = δ_2_ in (B) and corresponding K = 5 were chosen as final parameters.

However, we can learn δ and K simultaneously by changing δ, so as to seeking the optimal combination. This process is shown in Fig 7, δ defines the structure of data, we reveal this structure by scanning over M, each δ generates the curves of each Δ and K, for each δ, we identified the most distinguished structure with the highest Δ. Among all δ, we selected the most stable structure (staying the longest steps within the same cluster) with the highest stability measure. We recursively call the algorithm until the DICCCOL landmarks cannot be clustered furthermore, which finally resulted in a tree structure partition of data points. The multi-scale spectral clustering algorithm is formulated as follows.

### Input

The geodesic distance matrix G and the shortest path length P. Set T to be an empty tree.

### Output

A directed tree T. The leaf nodes contain all the landmarks.

### Algorithm

1. Initialize the Gaussian similarity matrix W_ij_(δ) according to the eq (3), where $\delta \;=\;[\delta_{min}:\frac{\delta_{max}-\delta_{min}}{size(G*P)}:\delta_{max}]$, δ_min_ = min((G+P)/2, δ_max_ = max((G+P)/2.
2. Compute Δ(M, δ) and K(M, δ) from δ_max_ to δ_min_ for each δ, beginning at M_0_ = 1, 2, 3…, ∞, until K(M_max_) = 1.
3. Find the maximum local maximum (Δ(M^1^, δ)) of Δ(M, δ) for each δ, among all δ, choose the most stable partitioning(K(M*, δ*)) which makes the stability measure α = M_b_—M_a_ maximum, Δ(M_b_, δ) and Δ(Ma,δ)are the next and previous local minimum of Δ(M^1^, δ).
4. Call the baseline spectral clustering algorithm with W = W(δ*) and K = K(M*,δ*), obtain K clusters.
5. For each cluster, call recursively this algorithm until the landmarks cannot be partitioned. Define the output as the *k*-th child of Tree T.

Here, size((G+P)/2) is the order of the square matrix Dis. We automatically derived the parameters δ and K simultaneously, corresponding to the size of length scales and the number of clusters within each scale. δ defines the structure of data, and we reveal this structure by scanning over M. δ and K are optimized at the same time, so as to seeking the optimal combination. In general, the larger δ is, the larger each cluster and Δ are expected to be. Thus, the corresponding cluster number K is smaller, so clustering in higher levels of the tree is obtained by using larger values of δ and each split is associated with a different δ.

### Identifying Fiber Bundles via Multi-scale Brain Networks

**Fig 8:**
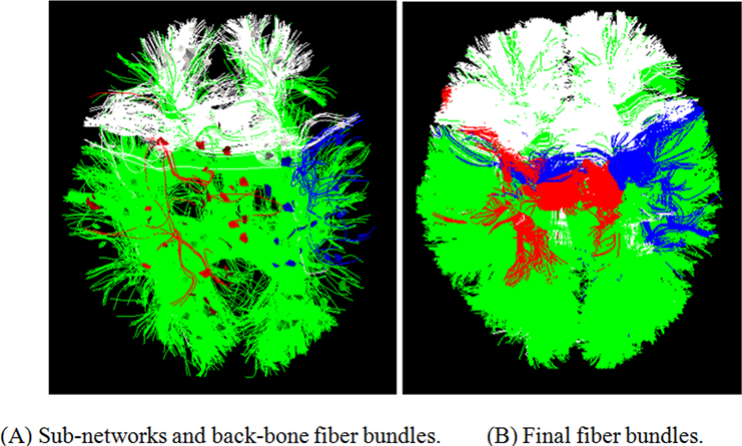
The two steps of identifying fiber bundles. (A) The backbone fiber bundles identified based on these multi-scale common sub-networks. (B) The remaining fibers were classified to these backbone bundles.

Based on these multi-scale and corresponding brain networks obtained in above section, we aim to identify multi-scale fiber bundles across individuals. First, we labeled these landmarks with the group number in multi-scale, and there are 1, 4, 11, 24, and 45 labels, respectively, in scale 1~5. We performed this step on one randomly chosen subject once, and then these labeled landmarks can be applicable to other subjects because the 358 cortical landmarks possess intrinsic correspondences. Then, we grouped all of the DTI-derived fibers connecting the pair of landmarks within each sub-network into the same fiber bundle, called backbone fiber bundles here, because they consist of the most consistent and representative fibers, as shown in Fig 8(A). Here is an illustrational example taken from scale 2, there are four groups of labels with different colors, and we identified the corresponding fiber bundles with the corresponding colors within each sub-network, as shown in Fig 8(A). Quantitatively, all backbone fibers at certain scale count for about 1%~13% of whole brain fibers, the percent is varying from scale 2 to scale 5. Finally, the traditional fiber clustering problem is converted into a fiber classification problem, in which the most popular mean closest distance [38,39] guides the fiber classification procedure, resulting in the final fiber bundles in Fig 8(B). We used a heuristic threshold for classification, the threshold was set to 4mm by averaging the some known fiber bundles generated from manually selected fiber bundles [40].

Conceptually, the major advantage of this methodology is that those consistent common landmarks across subjects and clustered multi-scale sub-networks define and form the reliable and corresponding backbone fiber bundles, which serve as the reliable basis for the following classification of fibers. Also, the classification step is not restricted to use the mean closest distance, and it can adopt any other effective features.

## Results/Discussion

### Multi-scale Common Brain Networks

**Fig 9:**
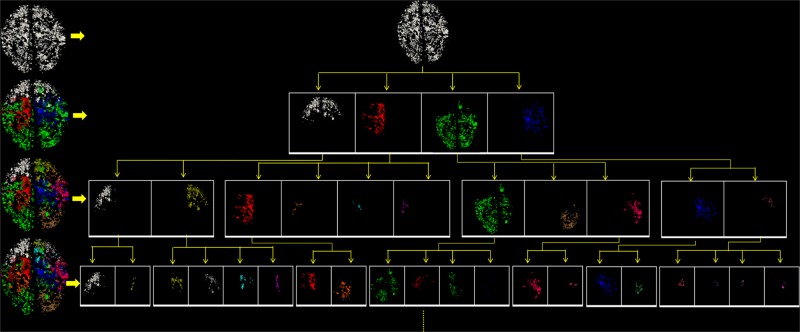
The tree structure of multi-scale division of brain network. From the top to the bottom, brain networks are divided into sub-networks, and each sub-network is divided again into the smaller sub-networks at the next layer. All sub-networks at the same layer are represented by different colors, and each new sub-network at the next scale is designated a new color. The numbers of sub-networks at each scale are 1, 4, 11, 24, and 45, respectively. For the convenience of visualization, the results from 5 randomly selected subjects were overlapped, and the sub-networks of the last scale are not shown due to limited space.

By applying the proposed methods on the DTI datasets 1, we generated the multi-scale common brain networks. As shown in Fig 9, the left column includes the reconstructed brain networks at each scale, and its sub-networks were shown in the right panel. Then if we check from the top to the bottom, each sub-network at one scale was divided again in the next scale, thus forming a hierarchical multi-scale tree structure. For the convenience of visual check, all the networks and sub-networks are represented as the overlay of 5 subjects at the same scale, and each brain sub-network was assigned with a different color. Altogether, the number of brain sub-networks at scales 1~5 are 1, 4, 11, 24, and 45, respectively. We can see that the landmarks were reasonably clustered into groups across individuals.

**Fig 10:**
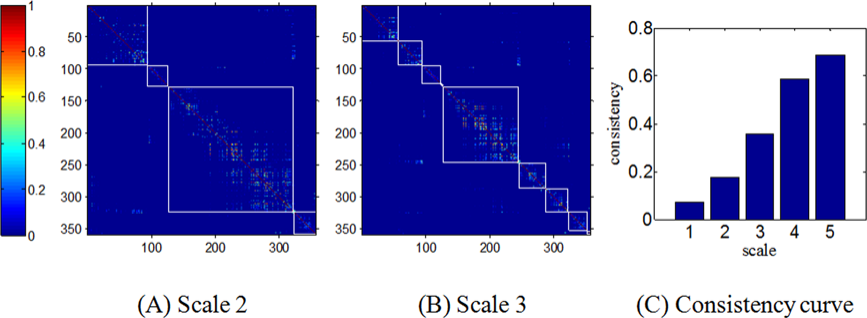
(A) and (B): The connectivity strength matrices of each sub-network at scale 2 and 3. (C) The consistency curve at all scales.

We defined the consistency within each sub-network as 0.5×*G*+0.5×*P*. Higher consistency means the landmarks within the same sub-network are closer (smaller geodesic distance) in cortex surface and have less hops of fiber connections from one landmark to another. In brief, higher consistency means more efficient information processing with less cost, like an economical small-world network [5]. Quantitatively, the consistencies within these sub-networks are stronger and stronger from scale 1 to 5. The matrix (0.5×*G*+0.5×*P*) in formula (3) can be regarded as a consistency measure, and Fig 10(A) and 10(B) show the matrix (0.5×*G*+0.5×*P*) of each sub-network in scale 2 and 3, from which we can see that the consistency is larger for the higher scale. It is also explained in (C), we computed mean (0.5×G+0.5×P) value for all sub-networks at the same scale. The consistency at different scales shows that the brain networks at higher scales have stronger consistency, which is quite reasonable and ensured by the multi-scale clustering algorithm intrinsically.

**Fig 11:**
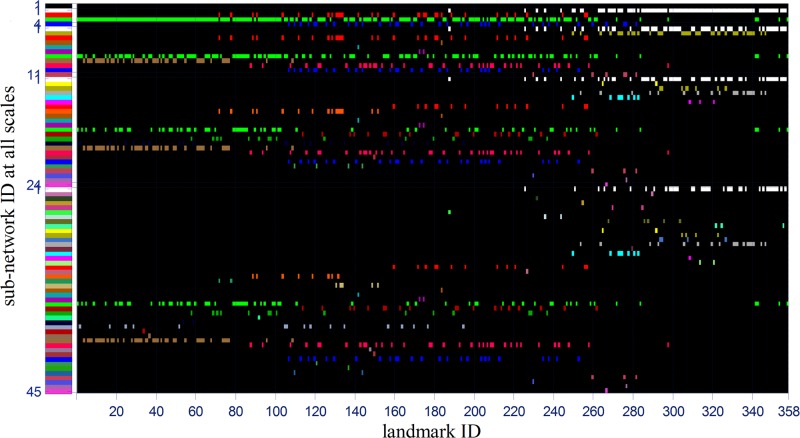
The look-up table between sub-network ID and its corresponding landmarks. The Y axis denotes the sub-network ID at all scales. From the top to the bottom, these IDs are 1, 1~4, 1~11, 1~24 and 1~45, respectively, and are shown by different colors. The X axis gives the landmark ID, 1~358. Then we can find the landmark ID that each sub-network includes by this table, that is, if we check from the left to the right at one row, all of the corresponding landmarks are represented by a colored rectangle. These colors are correspondent with those of all sub-networks in Fig 9.

**Fig 12:**
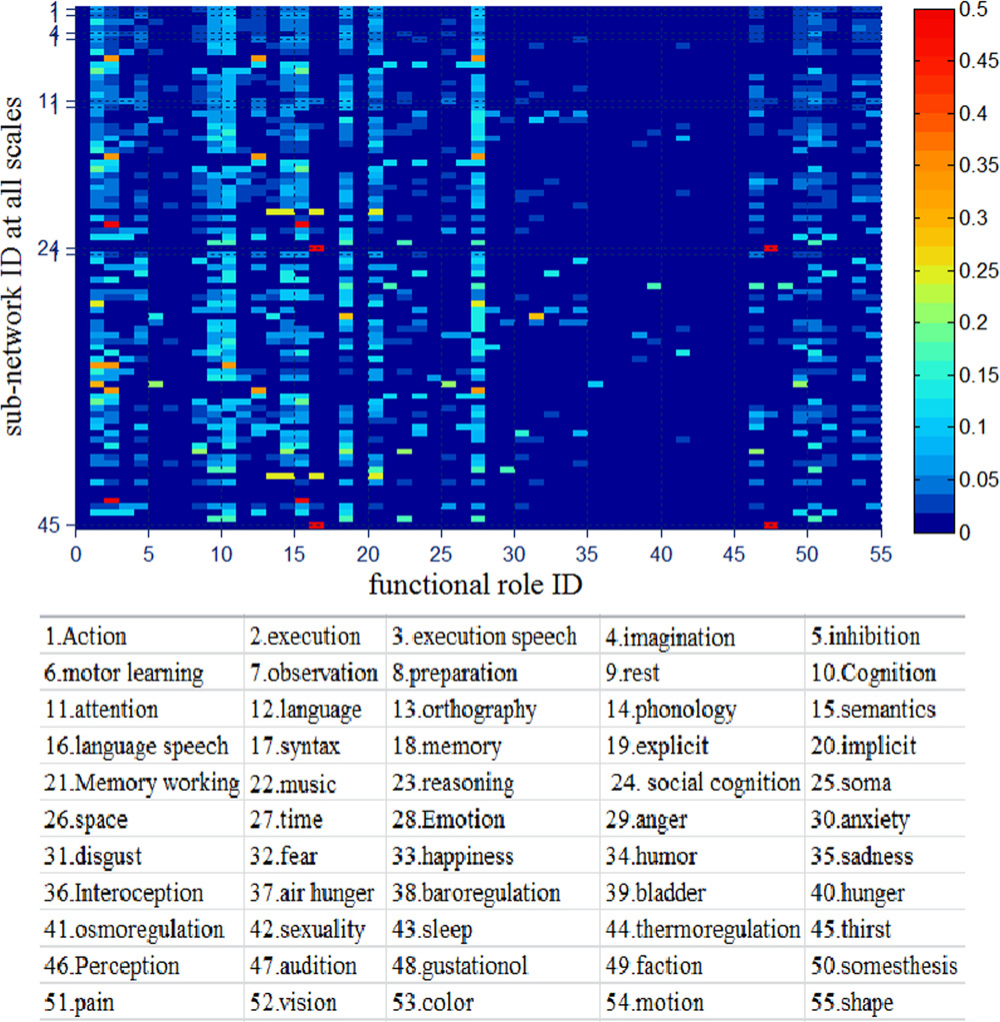
The percentage of functional roles of all sub-networks. The horizontal axis represents the 55 functional behavioral domains used in the BrainMap database [42], which are listed in the bottom, and the vertical axis represents sub-network ID. The right color bar shows the size of percentage.

The significance of these sub-networks is that they can be cross-validated by the existing discovered functional or structural sub-networks [41]. Specifically, we provided a look-up table as shown in Fig 11, by which we can find how many sub-networks each network includes and which DICCCOL landmarks each sub-network includes. Furthermore, we can infer the functional roles of each sub-network by counting the functional roles of all landmarks within a sub-network according to the recent meta-analysis results released in [41] based on the BrainMap database [42]. The percentage of each functional role of sub-network is shown in Fig 12. We can see that almost all the sub-network participate in some brain functional domains such as execution (2), attention (11), memory working (21) and emotion (28), etc. We will provide a detailed functional analysis and validation for sub-networks and fiber bundles in Section Results/Discussion.

### Explanation and Validation for Multi-scale Common Brain Networks

Taking the first sub-network at scale 2 as an example, we would validate it and its sub-networks at lower layers by existing prior knowledge. First, the left-most sub-network (the first sub-network) at scale 2 corresponded to frontal lobe by our rough visual check, then we also examined all its DICCCOL landmarks’ locations via transferring them to the Talairach space, found that there were only 5 out of 94 DICCCOL landmarks not belonging to frontal lobe, the three (landmark 265, 309, 321) belonging to limblic system and the two (314, 357) belonging to parietal lobe. By inquiring the functional role of this sub-network based on the previous resulting functional role table in Fig 12, we found its top 4 major functional roles are emotion (28), attention (11), cognition (10) and explicit (19), this is consistent with the common sense about frontal lobe, such as the anterior cingulate and medial prefrontal cortices play important roles in emotion process [43], the prefrontal cortices are activated in attention task [44].

Then we examined its sub-networks at the layer 3 in Fig 9, those are the left-most two sub-networks with gray and yellow color and derived from its parent node at the layer 2. We can see that its parent node was divided into the left and right parts, the left part’s top 4 functional roles are attention (11), emotion (28), semantics (15) and explicit (19) in that order, the right part’s top 4 functional roles are emotion (28), attention (11), cognition(10) and explicit (19), respectively. Their functional roles are reasonable given the existing researches about frontal lobe, such as that the left frontal lobe deals with language abilities (helps you think logically) while the right frontal lobe is generally more concerned with emotion (helps you think creatively) [45,46].

Finally we move to the right part’s four sub-networks at layer 4, which are the third, fourth, fifth, sixth sub-network respectively in Fig 9. Likewise, we can obtain their top functional roles as shown in Fig 12 by meta-analysis. The top one functional roles of the third, fourth, fifth and sixth sub-network are explicit (19), emotion (28), attention (11), and cognition (10), respectively. We can see that their parent node’s top four functional roles, which are emotion (28), attention (11), cognition (10) and explicit (19), are precisely divided into four parts, each of which exactly corresponds to the first major functional role of each sub-network at next layer. We examined the sixth sub-network whose DICCCOL landmarks were contained in 45 experiments of the BrainMap database [42], we searched its original fMRI experiment and the corresponding behavioral domain and found that most of them are located in the regions of cognition and execution speech. Also, this result is in agreement with other literature reports about the functional localizations of cognition and execution speech, e.g., in the Brodmann's area 32 [47–49], where some of the DICCCOLs of the sixth sub-network at layer 4 in Fig 9 concentrate on. We also examined the fifth sub-network whose functional roles mainly focus on attention, and especially on attention in language-processing task, it is quite reasonable given current literature reports, e.g., it was reported in [50] that parietal and inferior frontal sites were involved in attention in language-processing task. These results also demonstrated that a variety of cortical regions and networks exhibit strong functional diversity and heterogeneity [51–54], that is, a cortical region could participate in multiple functional domains/processes and a functional network might recruit various heterogeneous neuroanatomical areas.

### Prediction of Brain Networks in Individual Brains

**Fig 13:**
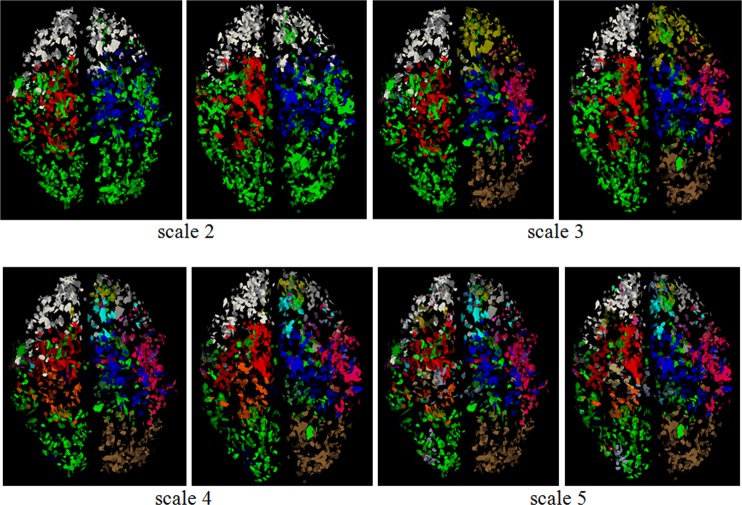
The predicted multi-scale common brain networks from nine different brains. For the convenience of visual check and comparison, the nine predicted brain network were overlaid in the left column of each panel, and the original model networks that correspond to the left-most column in Fig 9 are shown in the right column.

As a testing population, we used another independent DTI dataset 2 to predict the multi-scale brain networks. Since Fig 9 already maps the multi-scale organization of the 358 DICCCOLs into the hierarchical brain networks, the prediction procedure is straightforward under the guidance of the multi-scale network clustering results. Because of the intrinsic accurate correspondences established by the DICCCOLs, and each new subject’s DICCCOL is predicted via optimizing the location to minimize the difference between the model dataset and the subject to be predicted, meanwhile keeping the DICCCOLs in the models unchanged, the clustered network in the previous section can be readily used as the models to predict the tree structure for new individual brains, once the DICCCOL landmarks are predicted in the new subjects. Fig 13 shows the predicted brain networks from nine different subjects. For comparison, the original model networks in Fig 9 are put in the second column, and the first column shows the nine overlaid predicted brain networks. The color of each sub-network is the same as that of sub-network in Fig 9. It is evident that the predicted networks are very similar to those model networks. To represent the degree of overlap between each model and predicted sub-network, we also computed the Euclidean distances between representative landmarks in each model and predicted the sub-networks. The representative landmarks have the minimum mean distance from the other landmarks. As shown in Table 1, we can see that the distances between model and predicted sub-networks are relatively small, especially at higher scales. These results demonstrate the robustness of the multi-scale network construction framework, as well as the reproducibility of the DICCCOLs and the associated multi-scale brain networks.

**Table 1:** The Euclidean distance (mm) between the model and predicted sub-networks at each scale. The Euclidean distances between representative landmarks in each model and predicted the sub-networks were computed to represent the degree of overlap between each model and predicted sub-network.

	Sub-network ID	Distance	Average
**Scale 2**	1	21.3	19.1
	2	17.2	
	3	22.6	
	4	15.1	
**Scale 3**	1	22.7	15.5
	2	23.3	
	3	15.8	
	4	9.2	
	5	12.4	
	6	11.3	
	7	21.2	
	8	13.7	
	9	12.6	
	10	15.3	
	11	12.9	
**Scale 4**	1	21.7	10.6
	2	6.9	
	3	15.0	
	…	…	
	22	3.6	
	23	4.5	
	24	3.8	
**Scale 5**	1	20.2	9.3
	2	6.4	
	3	6.9	
	…	…	
	43	3.6	
	44	4.5	
	45	3.8	

### Application on Identification of Multi-scale Fiber Bundles

**Fig 14:**
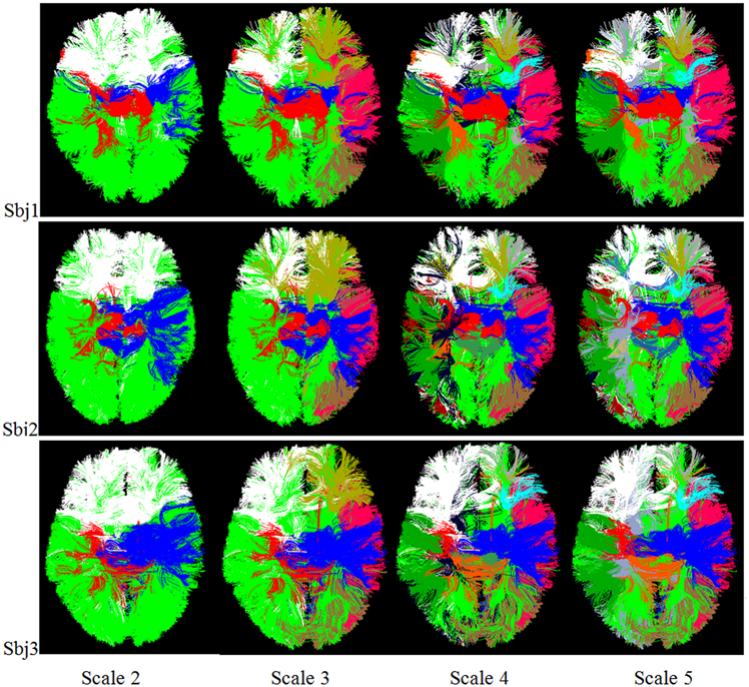
The multi-scale fiber bundles identified based on the multi-scale brain networks from three randomly selected subjects. Each corresponding fiber bundle across brains has the same color, and this color is also the same as that of the corresponding sub-network.

Based on the multi-scale and corresponding brain networks obtained in the above section, we clustered the fiber bundles according to the steps in section “Identifying Fiber Bundles via Multi-scale Brain Networks”, as shown in Fig 14. For the purpose of visual differentiation, different fiber bundles have different colors, and each corresponding fiber bundle in different brains has the same color. Furthermore, the color is same in the corresponding sub-network. It is evident that the multi-scale networks are able to guide the clustering of multi-scale fiber bundle, and these fiber bundles are more consistent at higher scales, in that the big fiber bundles were divided into more consistent bundle at the next scale. As a quantitative description, we computed the averaged Hausdorff distance of corresponding fiber bundles across subjects as [33]. Firstly, a representative fiber was selected as the center of fiber bundle in terms of Hausdorff distance. Then we averaged the distance of corresponding center fibers in different subjects at each scale. The averaged Hausdorff distance denotes the degree of consistency of corresponding fiber bundles among subjects, that is, the smaller it is, the higher consistency the fiber bundles have. Table 2 provided the averaged Hausdorff distances of common fiber bundles across subjects at each scale. We can see that the Hausdorff distances become relatively small at higher scale, indicating the accurate correspondence of fiber bundles across subjects. Finally, we averaged all the Hausdorff distances within the same scale, and the relatively small distances demonstrate that the fiber bundles at higher scales are more consistent.

**Table 2:** The averaged Hausdorff distance (mm) of common fiber bundles across subjects at multi-scales. Eg., the first value 1: 15.23 denotes that the averaged Hausdorff distance of 1# fiber bundle across subjects at scale2 is 15.23mm.

	Scale 2	Scale 3	Scale 4	Scale 5
**Fiber Bundle ID**	1: 15.23	1: 12.23	1:12.23	1: 12.23
	2: 7.18	2: 10.21	2: 3.58	2: 3.58
	3: 18.48	3: 7.18	3: 4.78	3: 4.78
	4: 8.45	4: 10.23	4: 10.47	4: 7.34
		5: 6.32	5: 3.45	5: 6.85
		6: 7.29	6: 5.57	6: 3.45
		7: 6.48	7: 5.32	7: 5.57
			8: 6.29	8: 5.32
			9: 7.34	9: 6.29
			10: 2.37	10: 3.30
				11: 4.53
				12: 2.37
**Average**	12.34	8.56	6.14	5.47

**Fig 15:**
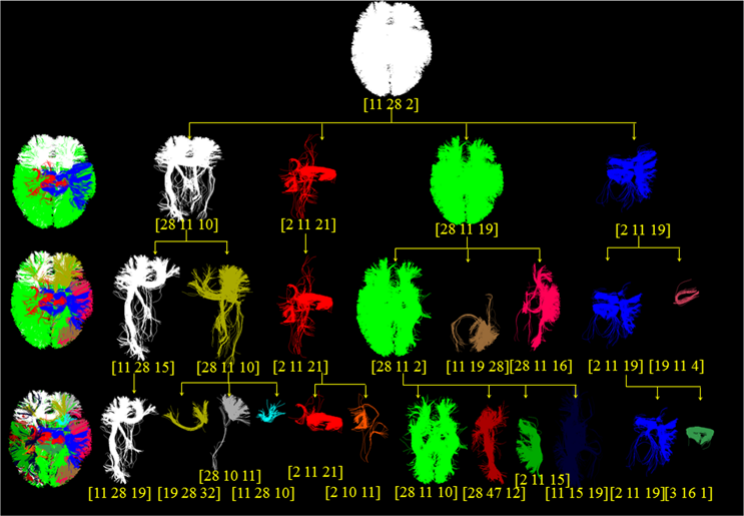
The multi-scale fiber bundles from one randomly selected subject and their top three major functional roles. From the top to the down, each fiber bundle was divided into several bundles at the next scale. The top three major functional roles are marked beneath each fiber bundle by the indices of 55 networks in Fig 11.

Also, we displayed the multi-scale divided fiber bundles and each bundle’s functional roles in Fig 15. It can be seen that fiber bundles at the next scale have more consistent shape than that at the former scale. This also demonstrated that sub-networks and corresponding fiber bundles are more consistent in higher scale. On the other side, based on the relationships between fiber bundles and sub-networks, we annotate each fiber bundle with the corresponding functional roles according to the information provided by Fig 12, and the top three major functional roles are marked beneath the fiber bundles. For example, the major 3 functions are attention (11), emotion (28) and execution (2), and the last fiber bundle at the lower right corner of the figure performs the functions of execution speech (3), language speech (16) and action (1). It will be very interesting to employ this information for tract-based analysis [39,55] in the future.

### Application on Schizophrenia Dataset

**Fig 16:**
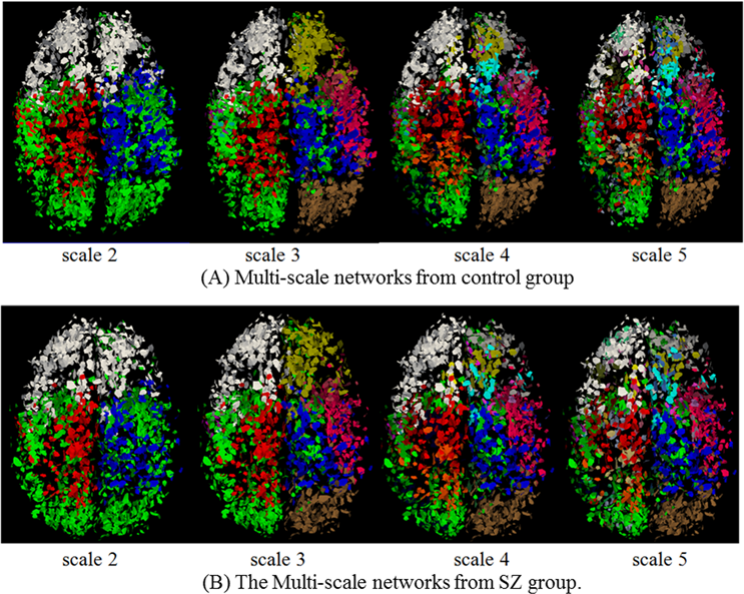
The predicted multi-scale common networks for SZ and control. The 10 control networks (A) and the 8 SZ networks (B) were overlaid to show, respectively.

**Fig 17:**
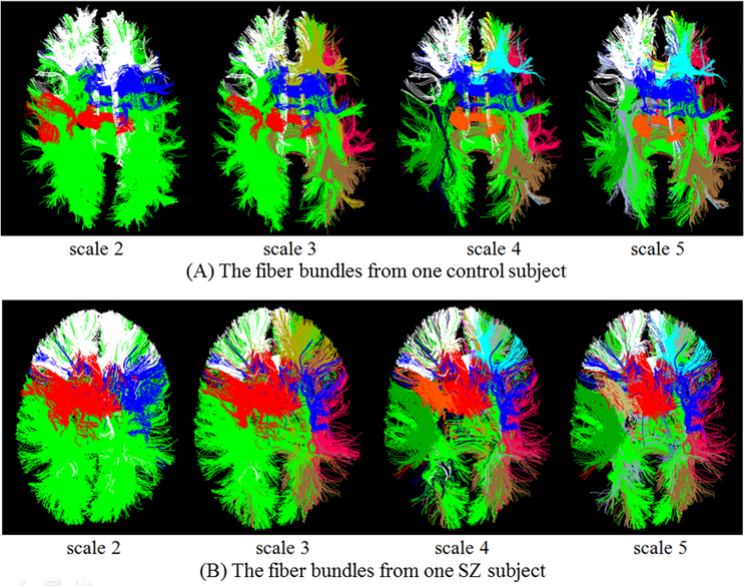
The predicted fiber bundles for SZ and control.

A variety of brain imaging studies of schizophrenia (SZ) [56–58] focused on the exploration of differences in volume, shape and fiber integrity, but results reported in the literature are very inconsistent [59]. By using the method in this paper, we first predicted the multi-scale brain network for SZ group and control group on the dataset 3. Then based on these multi-scale networks, we clustered the whole brain fibers into bundles at all scales, and finally, we compared the DTI-derived measurements of fractional anisotropy (FA) and mean diffusivity (MD) for fiber bundles between SZ patients and matched normal controls. Fig 16 shows the predicted multi-scale networks for SZ and control groups, which have the same corresponding colors with the previous ones. We randomly selected one subject from each group, respectively, to show the resulted fiber bundles (Fig 17). As we can see, the predicted multi-scale networks and fiber bundles are consistent with the original brain networks and fiber bundles. This result also suggests that our DICCCOL-based method can reliably, consistently and reproducibly identify the multi-scale brain networks and fiber bundles for new brains. For quantitative comparison, we counted the FA and MD values only along multi-scale common fiber bundles across all subjects, and performed two-tailed t-test. It was found that there is no statistical difference between SZ and control. There existed considerable papers supporting our result, although the existing more reports about schizophrenia claimed decreased FA with schizophrenia, in fact, there is no overwhelming point of view about SZ, these reports are very inconsistent from the location to direction of FA change, e.g., the popular point was that FA decreased in left frontal lobe and left temporal lobe [60], but there were still considerable researchers found there were no FA changes in these two areas and other locations [56,58] as our result.

However, it should be pointed out that although SZ and control exhibited no structural connectivity differences in terms of diffusion measure of fiber bundles based on the common DICCCOLs and their derived multi-scale network, we cannot exclude the possibility that there are structural connectivity differences in other brain networks and areas that are not covered by the DICCCOL nodes and their derived networks. Our interpretation of the current reported inconsistency of SZ’s structural connectivity is that the various definitions of node ROIs and brain networks (e.g., [56–59,61]) might be a determinant factor to account for such inconsistency. Thus, it is critically important to have principled approach to defining and constructing brain networks from neuroimaging datasets such that results and interpretations from different research labs can be fairly and effectively compared and integrated, and the DICCCOL system [24] and the work in this paper is an initial attempt in this direction.

## Conclusion

In this paper, we presented a novel computational framework for constructing multi-scale common brain networks across subjects based on an improved multi-scale spectral clustering method applied on DICCCOLs. Then these brain networks were used as models to predict multi-scale common brain networks for new subjects, and the results have demonstrated its reproducibility and robustness. As a direct application, we performed the fiber clustering based on these model networks and obtained multi-scale fiber bundles of the whole brain. This framework provides a novel and alternative way to identify fiber bundles for tract-based analysis. Furthermore, we applied the proposed method on a schizophrenic data, and the results verified its validity and reproducibility again.

The major novelties of this work include the intrinsically-established correspondences of those multi-scale networks and the optimal determination of the number of scales and the number of clusters in each scale. These two properties are preserved in the later fiber clustering procedure, and thus these networks have been applied to identify common and consistent fiber bundles. Furthermore, the functional roles of these brain networks and their corresponding fiber bundles can be inferred by the DICCCOL and meta-analysis, which is another contribution of this work. In the future, we plan to apply the proposed method and its derived multi-scale fiber bundles for tract-based analysis of DTI datasets of other brain diseases such as Alzheimer’s disease and Autism. It is conjectured that these common multi-scale structural brain networks can be potentially widely used as the basis for many other brain network modeling and analyses, such as modeling functional interactions and dynamics, in the future.
