# Correction: Construction of Multi-Scale Consistent Brain Networks: Methods and Applications

**DOI:** 10.1371/journal.pone.0130054

**Published:** 2015-06-03

**Authors:** Bao Ge, Yin Tian, Xintao Hu, Hanbo Chen, Dajiang Zhu, Tuo Zhang, Junwei Han, Lei Guo, Tianming Liu

The following information is missing from the Funding section: This study was supported by NSFC 61403243, the Fundamental Research Funds for the Central Universities from China (No. GK201402008) and Interdisciplinary Incubation Project of Learning Science of Shaanxi Normal University.
